# Supplementary material for: Unveiling the Bioactive Potential of the Invasive Jellyfish Phyllorhiza punctata Through Integrative Transcriptomic and Proteomic Analyses
Source: Biomolecules. 2025 Aug 4;15(8):1121. doi: 10.3390/biom15081121 (PMC12383608; doi:10.3390/biom15081121)
Supplement: Supplementary file 1 [file biomolecules-15-01121-s001.zip › Suplementary Material/FileS3/PEPFOLD4/XP_065063665_5413/clusters.html]

 PEP-FOLD clusters


Download archive of all models  here   

|  |  |  |
| --- | --- | --- |
| # | file | sOPEP |
| # cluster 1 - Size: 1 |  |  |
|  | XP\_065063665\_5413-model1 | -42.8964 |
| # cluster 2 - Size: 5 |  |  |
|  | XP\_065063665\_5413-model2 | -41.5492 |
|  | XP\_065063665\_5413-model2.1 | -39.6031 |
|  | XP\_065063665\_5413-model2.2 | -38.2817 |
|  | XP\_065063665\_5413-model2.3 | -37.5692 |
|  | XP\_065063665\_5413-model2.4 | -36.5628 |
| # cluster 3 - Size: 1 |  |  |
|  | XP\_065063665\_5413-model3 | -41.1044 |
| # cluster 4 - Size: 3 |  |  |
|  | XP\_065063665\_5413-model4 | -41.0812 |
|  | XP\_065063665\_5413-model4.1 | -36.3779 |
|  | XP\_065063665\_5413-model4.2 | -35.7341 |
| # cluster 5 - Size: 3 |  |  |
|  | XP\_065063665\_5413-model5 | -40.5804 |
|  | XP\_065063665\_5413-model5.1 | -38.0031 |
|  | XP\_065063665\_5413-model5.2 | -36.1714 |
| # cluster 6 - Size: 1 |  |  |
|  | XP\_065063665\_5413-model6 | -39.7846 |
| # cluster 7 - Size: 1 |  |  |
|  | XP\_065063665\_5413-model7 | -39.6317 |
| # cluster 8 - Size: 3 |  |  |
|  | XP\_065063665\_5413-model8 | -39.5484 |
|  | XP\_065063665\_5413-model8.1 | -36.2204 |
|  | XP\_065063665\_5413-model8.2 | -34.2236 |
| # cluster 9 - Size: 1 |  |  |
|  | XP\_065063665\_5413-model9 | -39.5346 |
| # cluster 10 - Size: 1 |  |  |
|  | XP\_065063665\_5413-model10 | -39.4573 |
| # cluster 11 - Size: 4 |  |  |
|  | XP\_065063665\_5413-model11 | -39.4142 |
|  | XP\_065063665\_5413-model11.1 | -37.853 |
|  | XP\_065063665\_5413-model11.2 | -37.3844 |
|  | XP\_065063665\_5413-model11.3 | -37.3395 |
| # cluster 12 - Size: 1 |  |  |
|  | XP\_065063665\_5413-model12 | -39.395 |
| # cluster 13 - Size: 2 |  |  |
|  | XP\_065063665\_5413-model13 | -38.6533 |
|  | XP\_065063665\_5413-model13.1 | -35.5242 |
| # cluster 14 - Size: 2 |  |  |
|  | XP\_065063665\_5413-model14 | -38.439 |
|  | XP\_065063665\_5413-model14.1 | -33.9633 |
| # cluster 15 - Size: 3 |  |  |
|  | XP\_065063665\_5413-model15 | -37.6315 |
|  | XP\_065063665\_5413-model15.1 | -35.9414 |
|  | XP\_065063665\_5413-model15.2 | -35.6851 |
| # cluster 16 - Size: 3 |  |  |
|  | XP\_065063665\_5413-model16 | -37.2093 |
|  | XP\_065063665\_5413-model16.1 | -36.9863 |
|  | XP\_065063665\_5413-model16.2 | -35.5076 |
| # cluster 17 - Size: 1 |  |  |
|  | XP\_065063665\_5413-model17 | -36.9113 |
| # cluster 18 - Size: 1 |  |  |
|  | XP\_065063665\_5413-model18 | -36.719 |
| # cluster 19 - Size: 4 |  |  |
|  | XP\_065063665\_5413-model19 | -36.604 |
|  | XP\_065063665\_5413-model19.1 | -35.665 |
|  | XP\_065063665\_5413-model19.2 | -34.206 |
|  | XP\_065063665\_5413-model19.3 | -32.0116 |
| # cluster 20 - Size: 1 |  |  |
|  | XP\_065063665\_5413-model20 | -36.5749 |
| # cluster 21 - Size: 3 |  |  |
|  | XP\_065063665\_5413-model21 | -36.5362 |
|  | XP\_065063665\_5413-model21.1 | -35.0625 |
|  | XP\_065063665\_5413-model21.2 | -34.765 |
| # cluster 22 - Size: 4 |  |  |
|  | XP\_065063665\_5413-model22 | -36.3141 |
|  | XP\_065063665\_5413-model22.1 | -35.8391 |
|  | XP\_065063665\_5413-model22.2 | -34.875 |
|  | XP\_065063665\_5413-model22.3 | -34.178 |
| # cluster 23 - Size: 1 |  |  |
|  | XP\_065063665\_5413-model23 | -36.2336 |
| # cluster 24 - Size: 2 |  |  |
|  | XP\_065063665\_5413-model24 | -35.9487 |
|  | XP\_065063665\_5413-model24.1 | -34.9467 |
| # cluster 25 - Size: 1 |  |  |
|  | XP\_065063665\_5413-model25 | -35.9391 |
| # cluster 26 - Size: 2 |  |  |
|  | XP\_065063665\_5413-model26 | -35.8423 |
|  | XP\_065063665\_5413-model26.1 | -33.0043 |
| # cluster 27 - Size: 3 |  |  |
|  | XP\_065063665\_5413-model27 | -35.6352 |
|  | XP\_065063665\_5413-model27.1 | -35.4014 |
|  | XP\_065063665\_5413-model27.2 | -35.298 |
| # cluster 28 - Size: 3 |  |  |
|  | XP\_065063665\_5413-model28 | -35.6052 |
|  | XP\_065063665\_5413-model28.1 | -34.9834 |
|  | XP\_065063665\_5413-model28.2 | -34.335 |
| # cluster 29 - Size: 2 |  |  |
|  | XP\_065063665\_5413-model29 | -35.4311 |
|  | XP\_065063665\_5413-model29.1 | -34.0707 |
| # cluster 30 - Size: 1 |  |  |
|  | XP\_065063665\_5413-model30 | -35.0212 |
| # cluster 31 - Size: 1 |  |  |
|  | XP\_065063665\_5413-model31 | -34.7602 |
| # cluster 32 - Size: 1 |  |  |
|  | XP\_065063665\_5413-model32 | -34.5832 |
| # cluster 33 - Size: 1 |  |  |
|  | XP\_065063665\_5413-model33 | -34.314 |
| # cluster 34 - Size: 2 |  |  |
|  | XP\_065063665\_5413-model34 | -34.1535 |
|  | XP\_065063665\_5413-model34.1 | -33.3483 |
| # cluster 35 - Size: 1 |  |  |
|  | XP\_065063665\_5413-model35 | -34.0323 |
| # cluster 36 - Size: 1 |  |  |
|  | XP\_065063665\_5413-model36 | -33.7348 |
| # cluster 37 - Size: 2 |  |  |
|  | XP\_065063665\_5413-model37 | -33.5104 |
|  | XP\_065063665\_5413-model37.1 | -33.4469 |
| # cluster 38 - Size: 2 |  |  |
|  | XP\_065063665\_5413-model38 | -33.4495 |
|  | XP\_065063665\_5413-model38.1 | -29.8489 |
| # cluster 39 - Size: 1 |  |  |
|  | XP\_065063665\_5413-model39 | -33.2 |
| # cluster 40 - Size: 1 |  |  |
|  | XP\_065063665\_5413-model40 | -33.048 |
| # cluster 41 - Size: 1 |  |  |
|  | XP\_065063665\_5413-model41 | -32.9926 |
| # cluster 42 - Size: 1 |  |  |
|  | XP\_065063665\_5413-model42 | -32.4818 |
| # cluster 43 - Size: 1 |  |  |
|  | XP\_065063665\_5413-model43 | -32.4624 |
| # cluster 44 - Size: 1 |  |  |
|  | XP\_065063665\_5413-model44 | -32.3575 |
| # cluster 45 - Size: 2 |  |  |
|  | XP\_065063665\_5413-model45 | -32.1771 |
|  | XP\_065063665\_5413-model45.1 | -31.8768 |
| # cluster 46 - Size: 1 |  |  |
|  | XP\_065063665\_5413-model46 | -31.8017 |
| # cluster 47 - Size: 1 |  |  |
|  | XP\_065063665\_5413-model47 | -31.5627 |
| # cluster 48 - Size: 2 |  |  |
|  | XP\_065063665\_5413-model48 | -31.0879 |
|  | XP\_065063665\_5413-model48.1 | -29.4651 |
| # cluster 49 - Size: 1 |  |  |
|  | XP\_065063665\_5413-model49 | -30.7594 |
| # cluster 50 - Size: 1 |  |  |
|  | XP\_065063665\_5413-model50 | -30.2926 |
| # cluster 51 - Size: 1 |  |  |
|  | XP\_065063665\_5413-model51 | -30.219 |
| # cluster 52 - Size: 1 |  |  |
|  | XP\_065063665\_5413-model52 | -30.1791 |
| # cluster 53 - Size: 2 |  |  |
|  | XP\_065063665\_5413-model53 | -30.1092 |
|  | XP\_065063665\_5413-model53.1 | -29.9422 |
| # cluster 54 - Size: 1 |  |  |
|  | XP\_065063665\_5413-model54 | -29.9488 |
| # cluster 55 - Size: 1 |  |  |
|  | XP\_065063665\_5413-model55 | -29.7656 |
| # cluster 56 - Size: 1 |  |  |
|  | XP\_065063665\_5413-model56 | -29.7179 |
| # cluster 57 - Size: 1 |  |  |
|  | XP\_065063665\_5413-model57 | -29.7174 |
| # cluster 58 - Size: 1 |  |  |
|  | XP\_065063665\_5413-model58 | -29.6418 |
| # cluster 59 - Size: 1 |  |  |
|  | XP\_065063665\_5413-model59 | -29.4654 |
| # cluster 60 - Size: 1 |  |  |
|  | XP\_065063665\_5413-model60 | -28.4341 |
|  |  |  |
